# Contraction of distance and duration production in autism spectrum disorder

**DOI:** 10.1038/s41598-019-45250-8

**Published:** 2019-06-19

**Authors:** Motoyasu Honma, Chihiro Itoi, Akira Midorikawa, Yasuo Terao, Yuri Masaoka, Takeshi Kuroda, Akinori Futamura, Azusa Shiromaru, Haruhisa Ohta, Nobumasa Kato, Mitsuru Kawamura, Kenjiro Ono

**Affiliations:** 1 Department of Physiology, Kyorin University School of Medicine, Tokyo, Japan; 2 Department of Psychology, Faculty of Letters, Chuo University, Tokyo, Japan; 3 Department of Physiology, Showa University School of Medicine, Tokyo, Japan; 4 Division of Neurology, Department of Medicine, Showa University School of Medicine, Tokyo, Japan; 5 Medical Institute of Developmental Disabilities Research, Showa University, Tokyo, Japan

**Keywords:** Human behaviour, Social behaviour

## Abstract

Autism spectrum disorder (ASD) presents certain hallmark features associated with cognitive and social functions, however, the ability to estimate self-generated distance and duration in individuals with ASD are unclear. We compared the performance of 20 ASD individuals with 20 typical developments (TDs) with respect to two tasks: (1) the drawing of a line of a specified distance (10 or 20 cm) and (2) waiting for a specified time (10 or 20 s). We observed that both the line distances and waiting times were substantially shorter in the ASD group than in the TD group. Furthermore, a trait of “attention to detail,” as measured by the Autism-Spectrum Quotient, correlated with some distance and duration productions observed in individuals with ASD. We suggest that attentional functions are related to the contraction of distance and duration in ASD.

## Introduction

The ability to understand and utilize distance and duration are vital for coordinated action control. Expressive control of space and time is a critical tool for information sharing and communication with others in social activities. Autism spectrum disorder (ASD) is a developmental disorder that begins early in childhood and lasts throughout life^1^. Initiating communication and responding to other people are challenging for individuals with ASD, and unawareness of social situations or blindness to those around them often results in confusion^2^. They exhibit atypical behavior associated with space and time, such as personal space^3^ and time management^4^.

Individuals with ASD have a marked impairment in the recognition of facial expressions^5^, gaze^6,7^, and language^8^, and these problems have been explained in terms of disruption of the social brain network, including the amygdala, superior temporal sulcus, fusiform area, and prefrontal cortex^9,10^. Furthermore, cerebro-cerebellar circuits, which bidirectionally connect the neocortex and cerebellum, are damaged in some individuals with ASD^11,12^, while there is a spectrum/heterogeneity in all individuals with an ASD^13^, suggesting that altered cerebellar connectivity may be linked to a lack of sociality.

Moreover, the perceptual level is atypical in ASD. People with ASD show a narrowing of spatial attention and are more apt to focus on details than on the whole^14,15^. Individuals with ASD are also less able to perceive the duration of a tone accurately in a comparison process^16^. However, although individuals with ASD have difficulty perceiving space and time accurately, it is not known whether they have difficulty performing within specified space and time parameters. In other words, although they show perceptual impairments, it is not known whether they show corresponding sensorimotor impairments.

When subjects with a typical development (TD) are asked to draw a line of a specific length, say, 10 cm, most can do this rather accurately without requiring a ruler. We might see similar results for time production (e.g., 10 s). However, if the impaired perceptual mechanism reflects the sensorimotor function in ASD, the individuals may show atypical productions of space and time. A further plausible conjecture is that the level of error may be related to the level of attention to detail because visual attention dilates the perceived duration of visual stimuli^17^. Furthermore, ASD shows a restricted range of attention/interests^18^. An attentional function in ASD may be related to a contraction in both space and time productions.

To test these ideas, we conducted behavioral experiments comparing the ability of individuals with ASD and TD to generate sensorimotor functions of specified distance and duration parameters. We used durations of 10 and 20 s because this allowed us to investigate consciously available behavior in the seconds range, unlike milliseconds or circadian scales. Previous research shows differences between TDs and individuals with ASD using such a time scale^19–21^. Furthermore, to identify autism-related factors associated with this ability, we conducted standardized tests of the ASD symptoms and compared the results with errors in the production of distance and duration.

## Results

**Figure 1:**
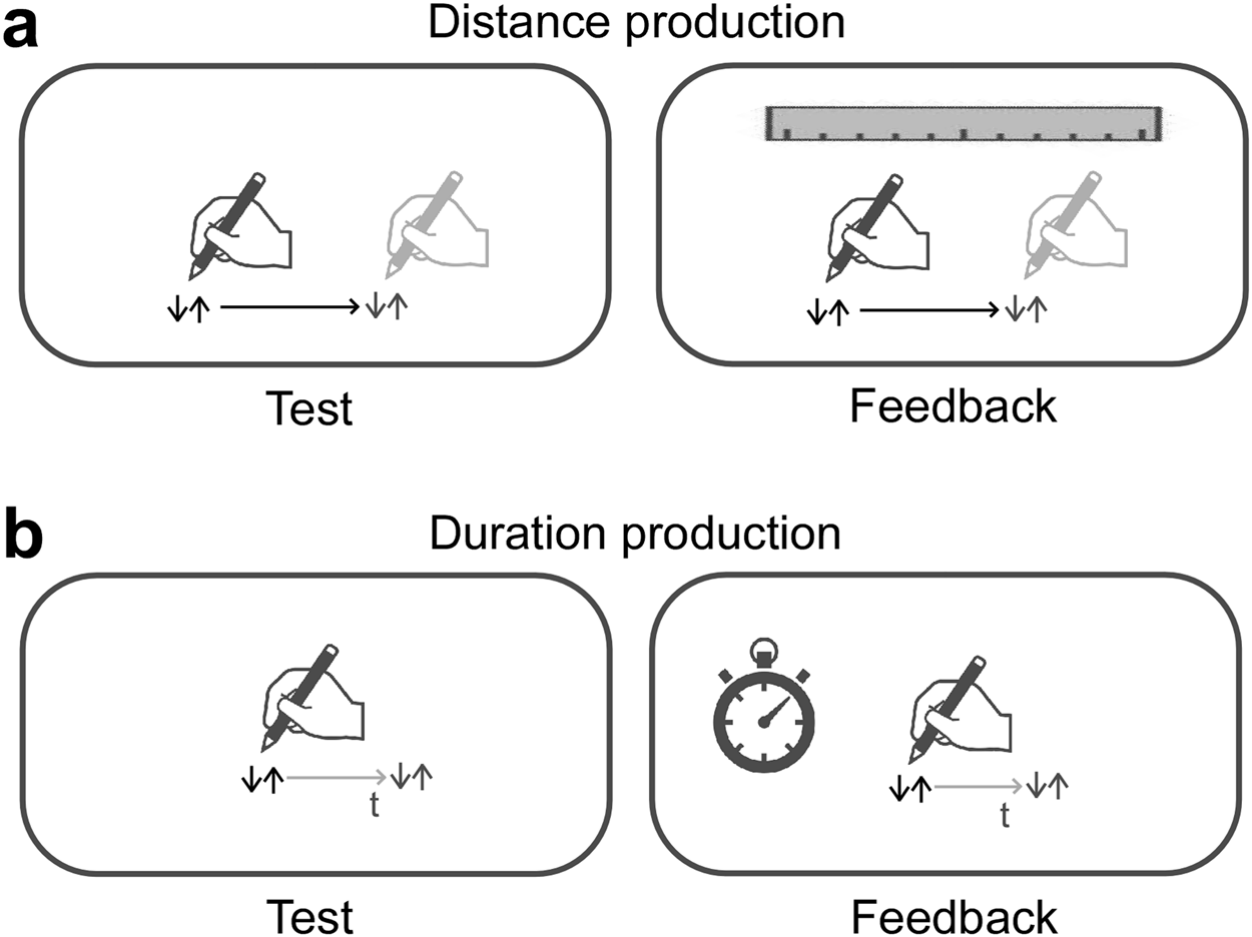
Illustration of trials. (**a**) The production task of distance. Left: A distance was produced by moving the pen for a specified distance. Right: The feedback task for distance production. A distance was produced by moving the pen for a specified distance by referencing the distance information on the “ruler” cue. (**b**) The production task of duration. Left: A duration was produced by tapping the tablet at the same position when a specified duration was felt to have elapsed. Right: The feedback task for duration production. A duration was produced by waiting for a specified duration to elapse by reference to the time information on the “clock” cue.

For the selected participants (see Methods), we conducted production tasks of distance and duration in both test and feedback sessions (Fig. 1). Furthermore, the severity of the ASD symptoms was measured using the Autism-Spectrum Quotient (AQ)^22,23^.

### AQ score

The scores on the AQ were higher for the ASD group than in the TD group (Table 1). The *t* tests revealed that the ASD group exhibited higher total scores than the TD group (*t*_38_ = 11.274, *p* < 0.0001) Similarly, the ASD group scored higher than the TD group in the social skills (*t*_38_ = 10.278, *p* < 0.0001), attention switching (*t*_38_ = 10.195, *p* < 0.0001), attention to detail (*t*_38_ = 5.344, *p* < 0.0001), communication skills (*t*_38_ = 7.786, *p* < 0.0001), and imagination domains (*t*_38_ = 6.399, *p* < 0.0001).

**Table 1:** Participant information.

	TD	ASD	*t*	*p*
Age (years)	28.0 (4.86)	29.8 (3.44)	1.350	0.185
Education (years)	15.3 (2.08)	15.1 (2.33)	0.358	0.722
Sex				
Female	4	4	—	—
Male	16	16	—	—
Hand dominance				
Right	20	20	—	—
Left	0	0	—	—
AQ score				
Total	15.2 (8.61)	37.9 (2.58)	11.274	<0.0001
Social skills	3.0 (2.00)	8.6 (1.39)	10.278	<0.0001
Attention switching	3.1 (2.27)	8.6 (0.82)	10.195	<0.0001
Attention to details	2.6 (1.15)	5.5 (2.14)	5.344	<0.0001
Communication skills	3.4 (2.52)	8.5 (1.61)	7.786	<0.0001
Imagination	3.2 (1.99)	6.6 (1.23)	6.399	<0.0001

TD: typical developments. ASD: individuals with autism spectrum disorder. Education: schooling history from elementary school. AQ: autism-spectrum quotient. The standard deviations are shown in parentheses.

### Production tasks

We attempted the tasks shown in the representative videos (Supplementary Table 1 and Supplementary Videos 1–16). Three samples (0.3%) of 960 trials (4 conditions [distance of 10 cm (“S10”) or 20 cm (“S20”), duration of 10 s (“T10”) or 20 s (“T20”)] × 3 repetitions × 2 sessions [test, feedback] × 40 participants) were excluded from the analysis because of missing data due to technical problems (1 TD in the “T20” condition of the test session; 1 TD in the “S10” and “T10” condition of the feedback session). Repeated measures analysis of variances (ANOVAs) with group as the between-subjects factor (TD and ASD) and session as the within-subjects factor (test and feedback) were performed for each of the 4 conditions (10/20 cm in distance and 10/20 s in duration).

**Figure 2:**
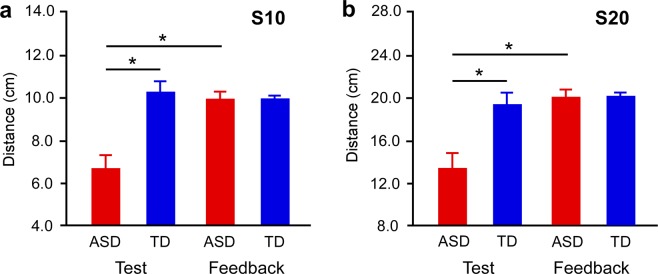
Distance production in test and feedback sessions. Distances produced in the (**a**) 10 cm and (**b**) 20 cm production tasks in the ASD and TD groups. Connecting brackets indicate statistically significant differences (*p* < 0.05). Error bars indicate SEM. ASD = individuals with autism spectrum disorder. TD = typical developments.

In the distance production task (Fig. 1a), the ASD group produced shorter distances than the TD group in the test session. In the “S10” condition (Fig. 2a), the ANOVA revealed main effects of group (*F*_1,37_ = 32.229, *p* < 0.0001, *η*^2^ = 0.466) and session (*F*_1,37_ = 19.503, *p* < 0.0001, *η*^2^ = 0.345), as well as their interaction (*F*_1,37_ = 33.901, *p* < 0.0001, *η*^2^ = 0.478). The post hoc tests showed that the distances produced by the ASD group (Supplementary Video 1) were shorter than those of the TD group (Supplementary Video 2) in the test session (*p* < 0.0001). In the ASD group, the distances produced in the test session were shorter than those in the feedback session (Supplementary Video 3) (*p* < 0.0001), while in the TD group, there was no difference between the test and feedback sessions (Supplementary Video 4). Similarly, in the “S20” condition (Fig. 2b), ANOVA revealed main effects of group (*F*_1,38_ = 39.118, *p* < 0.0001, *η*^2^ = 0.507) and session (*F*_1,38_ = 41.975, *p* < 0.0001, *η*^2^ = 0.525), as well as their interaction (*F*_1,38_ = 33.381, *p* < 0.0001, *η*^2^ = 0.468). The post hoc tests showed that the distances produced by the ASD group (Supplementary Video 5) were shorter than those of the TD group (Supplementary Video 6) in the test session (*p* < 0.0001), and in the ASD group the distances produced in the test session were shorter than those produced in the feedback session (Supplementary Video 7) (*p* < 0.0001), while in the TD group there was no difference between the test and feedback sessions (Supplementary Video 8).

**Figure 3:**
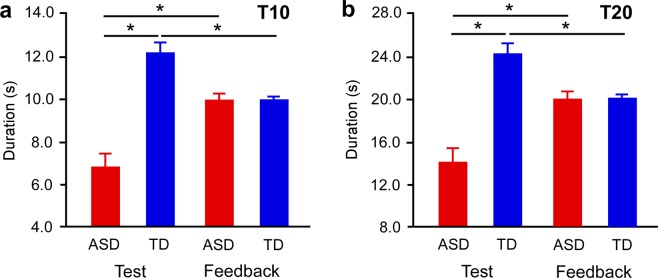
Duration production in test and feedback sessions. Durations produced in the (**a**) 10 s and (**b**) 20 s production tasks in the ASD and TD groups. Connecting brackets indicate statistically significant differences (*p* < 0.05). Error bars indicate SEM. ASD = individuals with autism spectrum disorder. TD = typical developments.

In the duration production task (Fig. 1b), the ASD group produced shorter durations than the TD group. In the “T10” condition (Fig. 3a), the ANOVA showed a main effect of group (*F*_1,37_ = 58.489, *p* < 0.0001, *η*^2^ = 0.613), no effect of session (*F*_1,37_ = 1.228, *p* = 0.275, *η*^2^ = 0.032), and a significant interaction (*F*_1,37_ = 54.328, *p* < 0.0001, *η*^2^ = 0.595). The post hoc tests showed that in the test session the duration produced by the ASD group (Supplementary Video 9) was shorter than that of the TD group (Supplementary Video 10) in the test session (*p* < 0.0001). In the ASD group, the duration produced in the test session was shorter than that in the feedback session (Supplementary Video 11) (*p* < 0.0001). In the TD group, the duration produced in the test session was longer than that in the feedback session (Supplementary Video 12) (*p* < 0.0001). Similarly, in the “T20” condition (Fig. 3b), the ANOVA showed a main effect of group (*F*_1,37_ = 65.536, *p* < 0.0001, *η*^2^ = 0.639), no effect of session (*F*_1,37_ = 0.010, *p* = 0.922, *η*^2^ = 0.001), and a significant interaction (*F*_1,38_ = 69.035, *p* < 0.0001, *η*^2^ = 0.651). The post hoc tests showed that in the test session the duration produced by the ASD group (Supplementary Video 13) was shorter than that of the TD group (Supplementary Video 14) (*p* < 0.0001). In the ASD group, the duration produced in the test session was shorter than that in the feedback session (Supplementary Video 15) (*p* < 0.0001). In the TD group, the duration produced in the test session was longer than that in the feedback session (Supplementary Video 16) (*p* < 0.0001).

As an additional analysis, we re-analyzed the difference between productions, using a ratio (by reducing S20/T20 to half), to test magnitude parameters as continuous variables. However, unpaired *t* tests showed no significant differences between the S10 and S20 tasks of distance in the ASD and TD groups. Similarly, the *t* tests showed no significant differences between the T10 and T20 tasks in the ASD and TD groups (Supplementary Fig. 1).

Furthermore, as a supplemental analysis of motor function, we analyzed the pen velocity in the distance tasks (Supplementary Fig. 2). In the S10 condition, the analysis showed that the velocity of the ASD group was greater than that of the TD group in the feedback session (*p* < 0.05), and in the ASD group the velocity of the feedback session was greater than that of the test session (*p* < 0.0001). In the S20 condition, the tests showed that the velocity of the ASD group was greater than that of the TD group in the feedback session (*p* < 0.001), and in the ASD group the velocity of the feedback session was greater than that of test session (*p* < 0.0001).

To confirm the effects of the medication, we added an analysis in the case of 7 individuals with ASD (ASD group = 13, TD group = 20; Supplementary Table 2). The tendency was similar to the non-reduced group in both ANOVA (Supplementary Table 3) and post hoc tests (Supplementary Table 4), suggesting that there was little effect of medication in the current study.

### Correlation between AQ scores and productions

**Figure 4:**
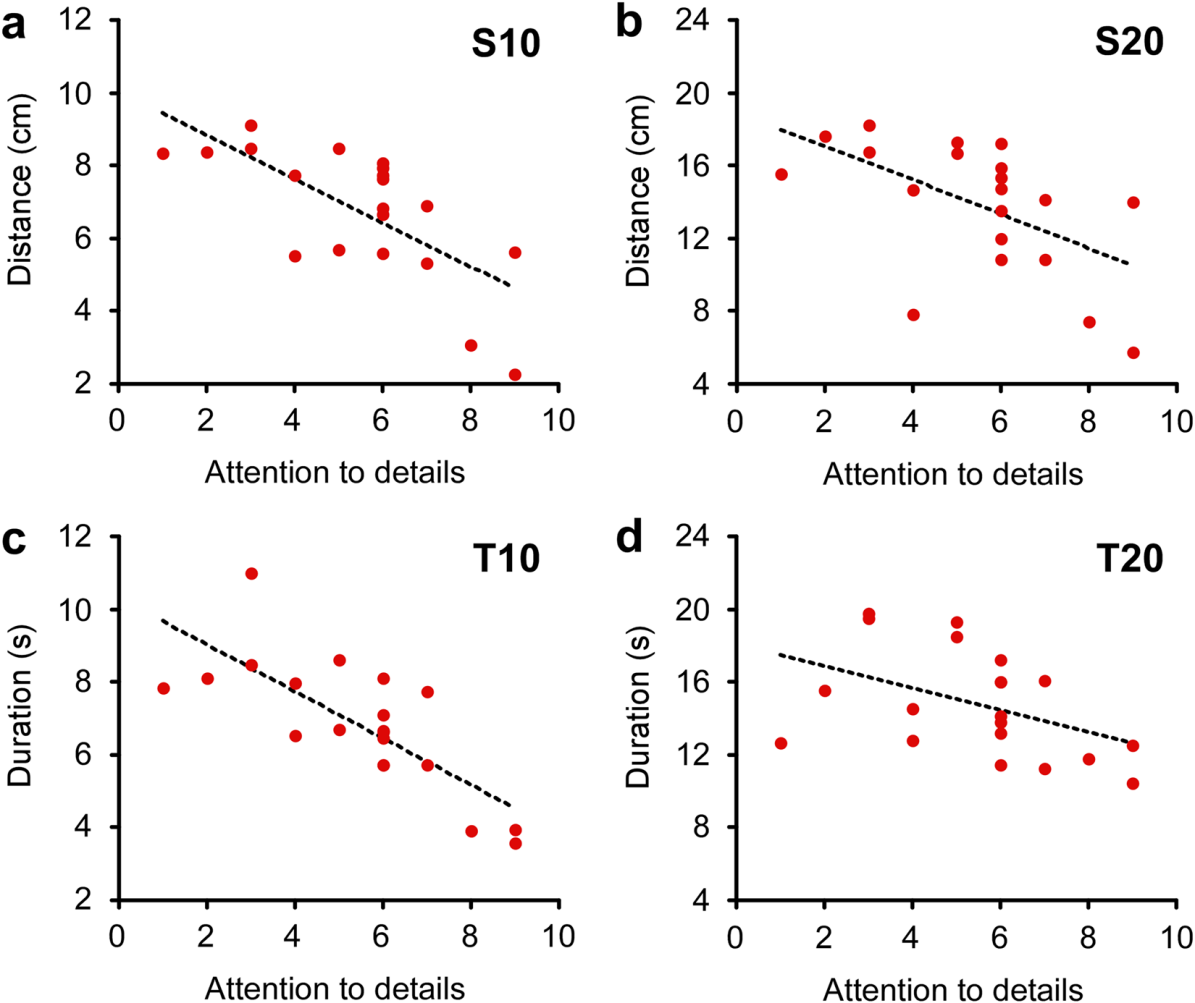
Correlation between attention to details AQ score and production of distance and duration in the ASD group. Each panel plots an across-individual scattergram between the attention to details sub score and production in the designated behavioral condition. (**a**) S10 (*r* = −0.702, *p* = 0.001), (**b**) S20 (*r* = −0.551, *p* = 0.012), (**c**) T10 (*r* = −0.761, *p* < 0.0001), and (**d**) T20 (*r* = −0.443, *p* = 0.049). The line indicates a linear regression fit.

The false discovery rate (FDR) was applied to the correlation analyses (significance *p* value on FDR = 0.008). In the ASD group, the “attention to detail” subscore of the AQ negatively correlated with the “S10” (Fig. 4a; *r* = −0.702, *p* = 0.001) and “T10” (Fig. 4c; *r* = −0.761, *p* < 0.0001), while the score did not correlate with “S20” (Fig. 4b; *r* = −0.551, *p* = 0.012) and “T20” (Fig. 4d; *r* = −0.443, *p* = 0.049) conditions. The total scores and other subscores showed no significant correlation for any of the conditions (all *r* < ± 0.35, *p* > 0.05, see Supplementary Table 5 and Supplementary Fig. 3). In addition, in the TD group, there were no significant correlations between any of the AQ scores and any of the conditions (all *r* < ± 0.20, *p* > 0.30, see Supplementary Table 6 and Supplementary Fig. 4). We also analyzed the correlation between the distance and duration data (significance *p* value on FDR = 0.025). The results revealed significant correlations between S10 and T10 (*r* = 0.828, *p* < 0.0001) and S20 and T20 (*r* = 0.781, *p* < 0.0001) in the ASD group (Supplementary Fig. 5), while there were no significant correlations in the TD group. These results indicate that the contractions in the distance and duration were interlocked in the ASD group.

## Discussion

The experiment in the test session confirmed a contraction of both distance and duration productions in individuals with ASD in comparison to individuals with TDs. In contrast, the produced distances and durations did not vary between the groups in the feedback session, in which a spatial or temporal cue was visually provided. Both ASDs and TDs could produce distances and durations with comparable accuracy in the presentation of spatial or temporal cues, indicating that ASDs could attend to the cues.

Heretofore, although it is known that a range from a perceptual aspect is contracted in individuals with ASD^17^, the current approach, which estimates self-generated distances and durations without cues, revealed that the range from an expressive aspect is also contracted in individuals with ASD. Furthermore, by analyzing the correlation between production using an assessment to estimate an atypical characteristic of and the severity of ASD, we found that the “attention to detail” scores in the AQ correlated with some contractions.

Studies of spatial perception have demonstrated connections from the visual to parietal and then to the prefrontal cortices^24^. In contrast, time perception may involve striatal networks interconnected with the hippocampus and parietal and prefrontal cortices^25^. Duration production is shortened in Parkinson’s disease (PD)^19,20^, while distance production is normal in PD^21^. In the case of PD, it is thought that the abnormal timing is due to the disruption of a striatum-centered neural network^20,26^; however, it has been unreported in ASD. In contrast, the cerebro-cerebellar circuits are damaged in some individuals with ASD^11–13^, and the cerebellum has been associated with sensorimotor functions including a control/modification of voluntary limb movements^27–29^. Higher brain dysfunction due to cerebellar deficits is known as cerebellar cognitive affective syndrome (CCAS)^30,31^. Individuals with ASD exhibit CCAS and also exhibit a dysmetria, which is an inability to stop a voluntary movement^32^. It remains possible that the contraction of distance and time duration in the current study may be caused by CCAS.

The atypical productions in ASD trended toward smaller, not larger, values for both distance and duration. Further, only the AQ subscores associated with “attention to detail” were correlated with the productions. We propose two possible mechanisms for the contraction observed in ASD. One is related to atypical attention. The weak central coherence theory suggests that people with ASD have difficulty combining individual elements into a meaningful whole, something people not on the spectrum do automatically^33,34^. Applied to our results, strong attention to detail may cause compression during production of distance and duration. Another explanation relates to atypical conceptualization. The literature on spatial association during number processing is dominated by the effects of spatial-numerical associations of response codes^35,36^. People with ASD may fail to accurately perform digital-analog conversions, or more specifically, they may underestimate the distance/duration during the conversion due to enhanced attention to small numbers. Furthermore, the current study revealed the correlation between distance and duration in the ASD group (Supplementary Fig. 5), while no correlation was noted in the TD group. This may show an interconnection between atypical distance and atypical duration productions in individuals with ASD. The contraction in ASD may occur at the level of representation of distance and duration, and it may actualize as an atypical sensorimotor functioning of ASD. In the analysis of motor function, the velocity analysis in the distance task showed that velocity was similar in both the feedback and test sessions in the TD group, while the velocity on feedback was faster than in the test session in the ASD group (Supplementary Fig. 2). Perhaps, atypical sensorimotor functioning in ASD patients may delay a process in the absence of a visual cue. This is an issue that needs to be addressed by future studies.

A limitation of our study was that we used restricted measurement ranges in terms of durations of 10 and 20 s and distances of 10 and 20 cm. With particular reference to the time scale, the processing level in the brain varies based on the time scales^37^. The cerebellum is mainly involved in sub-second processing^38,39^. However, a meta-analysis showed that the cerebellum also contributes to supra-second processing^40^. Had we used a few minutes or sub-second range in the duration task, different types of atypical contractions might have appeared. Indeed, the correlation between the “attention to detail” score and long scale productions (S20 and T20) were lower than the short scale productions (S10 and T10). These results may reflect different types of processing levels. Second, another subscale of AQ (social skills, attention switching, communication skills, and imagination) showed no relationship with the perceived distance and time duration. This may be dependent on the task demands. For instance, it appears that the subscores of the social skills, communication skills, and imagination reflect a social activity related to personal relationships. The subscore of attention switching may also be near a suppression function, and it may be correlated to a response inhibition task. Perhaps, the attention to detail subscore has a high affinity for behavioral tasks related to spatial and temporal representations. The third limitation is that we did not control for medication. Some individuals on medication were studied while medicated, because the majority of individuals with ASD suffered from restlessness and had difficulty managing the stylus pen under the drug *Off* condition. The number of subjects in our study was also too small to reveal a medication effect, while an additional analysis, removing 7 individuals on medication, showed that the tendency in the reduced group was similar to that of the non-reduced group. A sufficient number of samples in the *On*/*Off* states is required to determine the effects of medication because antipsychotics inhibit inappropriate behavior but can adversely affect various cognitive functions^41^. Finally, the current study investigated the spatial/temporal characteristics in ASD from the aspects of production not perception. Individuals with ASD show a restricted range of attention/interests^18^. A passive task, such as a bisection task or attentional task, may reveal further insights into the impairment of production in individuals with ASD.

We found that individuals with ASD under-produced distances and time durations. This is a novel characteristic of ASD. Correct production of distance and duration may play a mediating role in social interactions, and deviations may participate in disrupting the communication ability in ASD. This easy-to-use experimental paradigm may be helpful in providing a diagnosis of ASD. Furthermore, a modification towards normalization of distance and duration production may contribute to an improvement of social communication ability in individuals with ASD.

## Methods

### Standard protocol approvals registration, and patient consent

This study was approved by the Ethics Committees of Showa University Karasuyama Hospital (clinical trial identifier number: B-2015-021) and was conducted according to the Principles of the Declaration of Helsinki. All participants provided written informed consent.

### Participants

In a case-controlled study design, the sample size was determined on the basis of effect sizes obtained in previous studies related to cognitive function^21,42^. The diagnosis of ASD was based on a consensus reached by two psychiatrists according to the criteria of the Diagnostic and Statistical Manual of Mental Disorders (DSM-IV-TR). Two detailed interviews were conducted independently by a clinical psychologist. Criteria included the participants’ developmental history, present illness, past history, and family history. The initial sample size included 40 individuals. From this group, 20 individuals uncomplicated with other diseases (Attention-Deficit Hyperactivity Disorder) were selected to participate in this study. Furthermore, Wechsler Adult Intelligence Scale-III^43^ (Full-Scale IQ: average [110.8], range [91 to 135]; Verbal IQ: average [115.7], range [99 to 131]; Performance IQ: average [102.2], range [90 to 129]), to ensure the high functionality of individuals with ASD. Seven were routinely prescribed antidepressant medications, and participated in behavioral experiments in the drug *On* condition, under which medicines were being administered. Twenty TDs were randomly selected by matching the average age, sex distribution, and schooling history of the ASDs. Therefore, we conducted the experiment on the basis that the IQs of the TDs are near average on a common distribution. There were no significant differences in education between the TD and ASD groups, and all participants were right-handed (Table 1).

### Clinical assessment

The severity of the ASD symptoms was measured using the Japanese version of the AQ^22,23^. This uses 50 questions to measure the extent of autistic traits in adults. These cover five domains associated with the autism spectrum, namely, social skills, communication skills, imagination, attention to details, and attention switching. Each question has possible responses of “definitely agree,” “slightly agree,” “slightly disagree,” or “definitely disagree,” and the responses are assigned a score of 1 if they slightly or definitely indicate autism, and 0 otherwise. Thus, higher scores indicate greater severity of autistic symptoms. Eighty percent of those diagnosed with autism, or a related disorder, score 32 or higher. We measured AQ roughly three months before the behavioral experiments.

### Behavioral measurement

Participants produced a specified distance or duration with their dominant hand (right) by holding a stylus pen above an electronic tablet (Intuos4 Extra Large, WACOM Corporation, Saitama, Japan; spatial precision ± 0.25 mm, sampling rate 200 points/s, screen size 488 mm × 305 mm, frame size 623 mm × 462 mm). At the beginning of each trial, the distance or duration to be produced was verbally stated by the experimenter, and the start of the production task was self-paced for each participant. The participants hovered the stylus over a point when moving the stylus towards the right. The distance and time duration were measured between the two points.

In the production task of distance (Fig. 1a), the participants were asked to move the pen to the right for a distance of 10 cm (“S10,” Supplementary Videos 1 and 2) or 20 cm (“S20,” Supplementary Videos 5 and 6). In the production task of duration (Fig. 1b), participants were asked to tap the tablet with a pen to start, wait for 10 s (“T10,” Supplementary Videos 9 and 10) or 20 s (“T20,” Supplementary Videos 13 and 14) condition, and then tap the tablet again at the end of the interval. During the test sessions, participants completed the task without any cues. In the feedback sessions, a ruler cue for the distance task (Fig. 1a, Supplementary Videos 3, 4, 7, 8) and a clock cue for the duration task (Fig. 1b, Supplementary Videos 11, 12, 15, 16) were provided in each trial. The ruler cue was a drawing of a ruler with its scale in millimeters and a maximum value of 30 cm. The clock cue was a drawing of an analog clock with its dial scaled in seconds with a maximum value of 60 s. The second hand moved in a smooth rotation as in a real clock. The trials were repeated three times per condition (total 24 trials per participant), and the 3 data points were averaged for analysis. The order was randomized among the test sessions, while the feedback session was always conducted last.

As a supplemental analysis of motor function, we analyzed the velocity of the distance task. The velocity was calculated by dividing distance (cm) by duration (s) in both the test and feedback conditions. The tasks in the feedback condition were simpler than those in the test session, so we assumed that the feedback session was appropriate for assessing sensorimotor function.

### Statistical analysis

Repeated measures ANOVA and post hoc tests with Bonferroni correction were performed for the behavioral data, and unpaired *t* tests were performed for clinical assessments and for medication effects. The distance and duration data were analyzed separately. In addition, the 10/20 cm and 10/20 s conditions were analyzed independently. The results are shown as the mean ± standard error of mean and effect size (eta squared, *η*^2^). Furthermore, the FDR was applied to the correlation analyses.

## Supplementary information

Supplementary Video 1

Supplementary Video 2

Supplementary Video 3

Supplementary Video 4

Supplementary Video 5

Supplementary Video 6

Supplementary Video 7

Supplementary Video 8

Supplementary Video 9

Supplementary Video 10

Supplementary Video 11

Supplementary Video 12

Supplementary Video 13

Supplementary Video 14

Supplementary Video 15

Supplementary Video 16

Supplementary Info
