# Supplementary material for: Contraction of distance and duration production in autism spectrum disorder
Source: Sci Rep. 2019 Jun 19;9:8806. doi: 10.1038/s41598-019-45250-8 (PMC6584662; doi:10.1038/s41598-019-45250-8)
Supplement: Supplementary file 17 — Supplementary Info [file 41598_2019_45250_MOESM17_ESM.pdf]

Supplementary Information

Contraction of distance and duration production in autism spectrum disorder

Motoyasu Honma[1], Chihiro Itoi[2], Akira Midorikawa[2], Yasuo Terao[1], Yuri Masaoka[3], Takeshi Kuroda[4], Akinori Futamura[4], Azusa Shiromaru[4], Haruhisa Ohta[5], Nobumasa Kato[5], Mitsuru Kawamura[4], and Kenjiro Ono[4]

[1]Department of Physiology, Kyorin University School of Medicine, Tokyo, Japan.

[2]Department of Psychology, Faculty of Letters, Chuo University, Tokyo, Japan.

[3]Department of Physiology, Showa University School of Medicine, Tokyo, Japan.

[4]Department of Neurology, Showa University School of Medicine, Tokyo, Japan.

[5]Medical Institute of Developmental Disabilities Research, Showa University, Tokyo, Japan.

Correspondence to: Motoyasu Honma (mhonma@ks.kyorin-u.ac.jp) +81-422-47-5511, Department of Physiology, Kyorin University School of Medicine, 6-20-2 Shinkawa, Mitaka-shi, Tokyo 181-8611, Japan and Kenjiro Ono (onoken@med.showa-u.ac.jp) +81 3 3784 8781, Department of Neurology, Showa University School of Medicine 1-5-8 Hatanodai, Shinagawa-ku, Tokyo 142-8666, Japan.

Supplementary Tables 1-6

Supplementary Figures 1-5

Supplementary Videos 1-16

Supplementary Table 1. List of the representative videos.

File name	Condition	Session	Subject
S-Video 1	S10	test	ASD
S-Video 2	S10	test	TD
S-Video 3	S10	feedback	ASD
S-Video 4	S10	feedback	TD
S-Video 5	S20	test	ASD
S-Video 6	S20	test	TD
S-Video 7	S20	feedback	ASD
S-Video 8	S20	feedback	TD
S-Video 9	T10	test	ASD
S-Video 10	T10	test	TD
S-Video 11	T10	feedback	ASD
S-Video 12	T10	feedback	TD
S-Video 13	T20	test	ASD
S-Video 14	T20	test	TD
S-Video 15	T20	feedback	ASD
S-Video 16	T20	feedback	TD

Supplementary Table 2. Participant details with the exception of 7 individuals with ASD on medication.

	TD	ASD	*t*	*p*
Age (years)	28.0 (4.86)	29.8 (3.44)	1.350	0.185
Education (years)	15.3 (2.08)	15.1 (1.75)	0.358	0.722
Sex				
Female	4	4	-	-
Male	16	9	-	-
Hand dominance				
Right	20	13	-	-
Left	0	0	-	-
AQ score				
Total	15.2 (8.61)	38.8 (2.59)	9.551	< 0.0001
Social skills	3.0 (2.00)	8.1 (1.14)	9.541	< 0.0001
Attention switching	3.1 (2.27)	8.6 (0.87)	8.338	< 0.0001
Attention to detail	2.6 (1.15)	5.1 (2.18)	5.290	< 0.0001
Communication skills	3.4 (2.52)	8.0 (0.99)	7.469	< 0.0001
Imagination	3.2 (1.99)	6.6 (1.21)	5.912	< 0.0001

TD: typical developments. ASD: individuals with autism spectrum disorder. Education: schooling history from elementary school. AQ: autism-spectrum quotient. The standard deviations are shown in parentheses.

Supplementary Table 3. ANOVA for the version with the exception of 7 individuals with ASD on medication.

Condition	Effect	df	*F*	*p*	*η2*
S10	group	1, 30	23.016	< 0.0001	0.434
	session	1, 30	14.281	0.001	0.323
	interaction	1, 30	25.142	<0.0001	0.456
S20	group	1, 31	37.131	<0.0001	0.545
	session	1, 31	39.575	<0.0001	0.561
	interaction	1, 31	31.086	<0.0001	0.501
T10	group	1, 30	47.227	<0.0001	0.612
	session	1, 30	1.227	0.277	0.039
	interaction	1, 30	43.763	<0.0001	0.593
T20	group	1, 30	49.217	<0.0001	0.621
	session	1, 30	0.001	0.998	0.001
	interaction	1, 30	45.462	<0.0001	0.602

Supplementary Table 4. Post hoc test for the version with the exception of 7 individuals with ASD on medication.

Condition	Session	TD	ASD	*p*
S10	test	10.53 (2.23)	6.88 (1.75)	< 0.0001
	feedback	10.07 (0.12)	10.19 (0.23)	0.078
S20	test	19.78 (2.39)	14.03 (3.12)	< 0.0001
	feedback	20.14 (0.28)	19.88 (0.94)	0.260
T10	test	12.25 (2.61)	6.73 (1.56)	< 0.0001
	feedback	10.02 (0.29)	9.85 (0.36)	0.141
T20	test	24.89 (4.58)	14.93 (2.95)	< 0.0001
	feedback	20.02 (0.27)	19.81 (0.46)	0.121

Supplementary Table 5. Correlations between the AQ scores and the production of distance and duration in the ASD group.

	S10		S20		T10		T20	
AQ scores	*r*	*p*	*r*	*p*	*r*	*p*	*r*	*p*
Social Skills	0.208	0.379	0.188	0.427	0.290	0.214	0.176	0.457
Attention Switching	0.443	0.051	0.293	0.210	0.356	0.123	0.027	0.909
Attention to detail	-0.702	0.001	-0.551	0.012	-0.761	0.0001	-0.443	0.049
Communication Skills	0.308	0.187	0.358	0.121	0.251	0.287	0.142	0.549
Imagination	-0.163	0.491	-0.293	0.209	-0.034	0.887	-0.211	0.373
Total Score	-0.167	0.481	-0.140	0.557	-0.172	0.469	-0.214	0.365

Supplementary Table 6. Correlations between the AQ scores and the production of distance and duration in the TD group.

	S10		S20		T10		T20	
AQ scores	*r*	*p*	*r*	*p*	*r*	*p*	*r*	*p*
Social Skills	-0.177	0.454	0.243	0.302	-0.186	0.433	-0.254	0.294
Attention Switching	-0.241	0.306	0.037	0.875	-0.134	0.574	-0.113	0.646
Attention to detail	-0.116	0.625	0.215	0.362	-0.158	0.506	-0.258	0.287
Communication Skills	-0.118	0.619	0.115	0.629	-0.094	0.693	-0.118	-0.632
Imagination	-0.080	0.738	0.204	0.387	-0.013	0.956	-0.021	0.933
Total Score	-0.173	0.465	0.176	0.458	0.130	0.585	-0.170	0.488

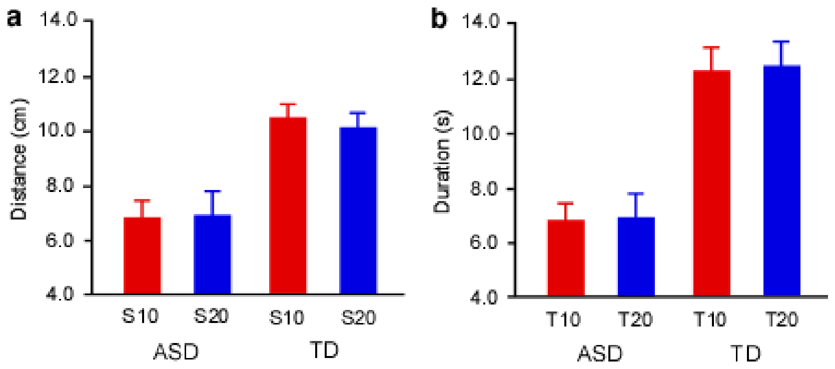

Supplementary Figure 1. The ratio analysis for distance and duration productions. We re-analyzed the difference between productions, using a ratio (by reducing the S20/T20 to half), to test the magnitude of the parameters as continuous variables. **a)** Distance production. Unpaired *t* tests showed no significant differences between the S10 and S20 tasks in the ASD and TD groups. **b)** Duration production. Similarly, the *t* tests showed no significant differences between the T10 and T20 tasks in the ASD and TD groups. TD: typical developments. ASD: individuals with autism spectrum disorder.

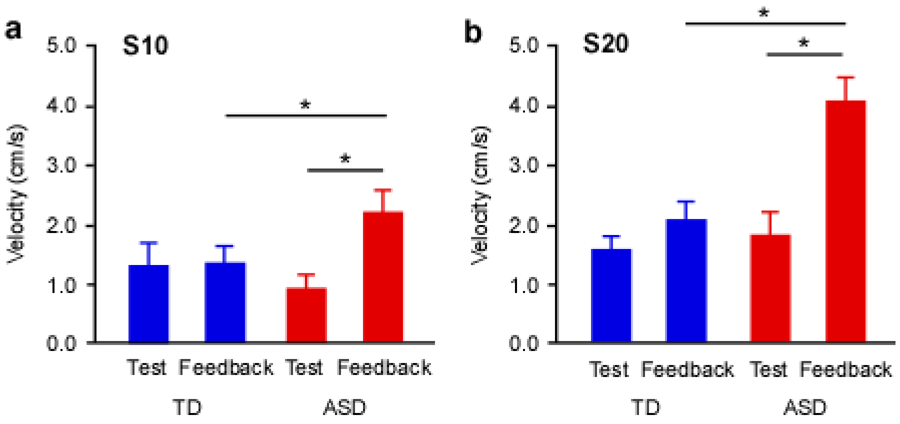

Supplementary Figure 2. Velocity in the distance production. For the velocity in the S10 condition (**a**), the ANOVA revealed main effects of group ($F_{1,38} = 0.548$, $p = 0.464$, $\eta^2 = 0.014$) and session ($F_{1,38} = 17.076$, $p < 0.0001$, $\eta^2 = 0.310$), as well as their interaction ($F_{1,38} = 13.087$, $p < 0.001$, $\eta^2 = 0.256$). The post hoc tests showed that the velocity of the ASD group was greater than that of the TD group in the feedback session ($p < 0.05$), and in the ASD group the velocity of the feedback session was greater than that of the test session ($p < 0.0001$). For the velocity in the S20 condition (**b**), the ANOVA revealed main effects of group ($F_{1,37} = 8.371$, $p < 0.01$, $\eta^2 = 0.014$) and session ($F_{1,37} = 9.908$, $p < 0.005$, $\eta^2 = 0.211$), as well as their interaction ($F_{1,37} = 12.034$, $p < 0.001$, $\eta^2 = 0.185$). The post hoc tests showed that the velocity of the ASD group was greater than that of the TD group in the feedback session ($p < 0.001$), and in the ASD group the velocity of the feedback session was greater than that of the test session ($p < 0.0001$). TD: typical developments. ASD: individuals with autism spectrum disorder.

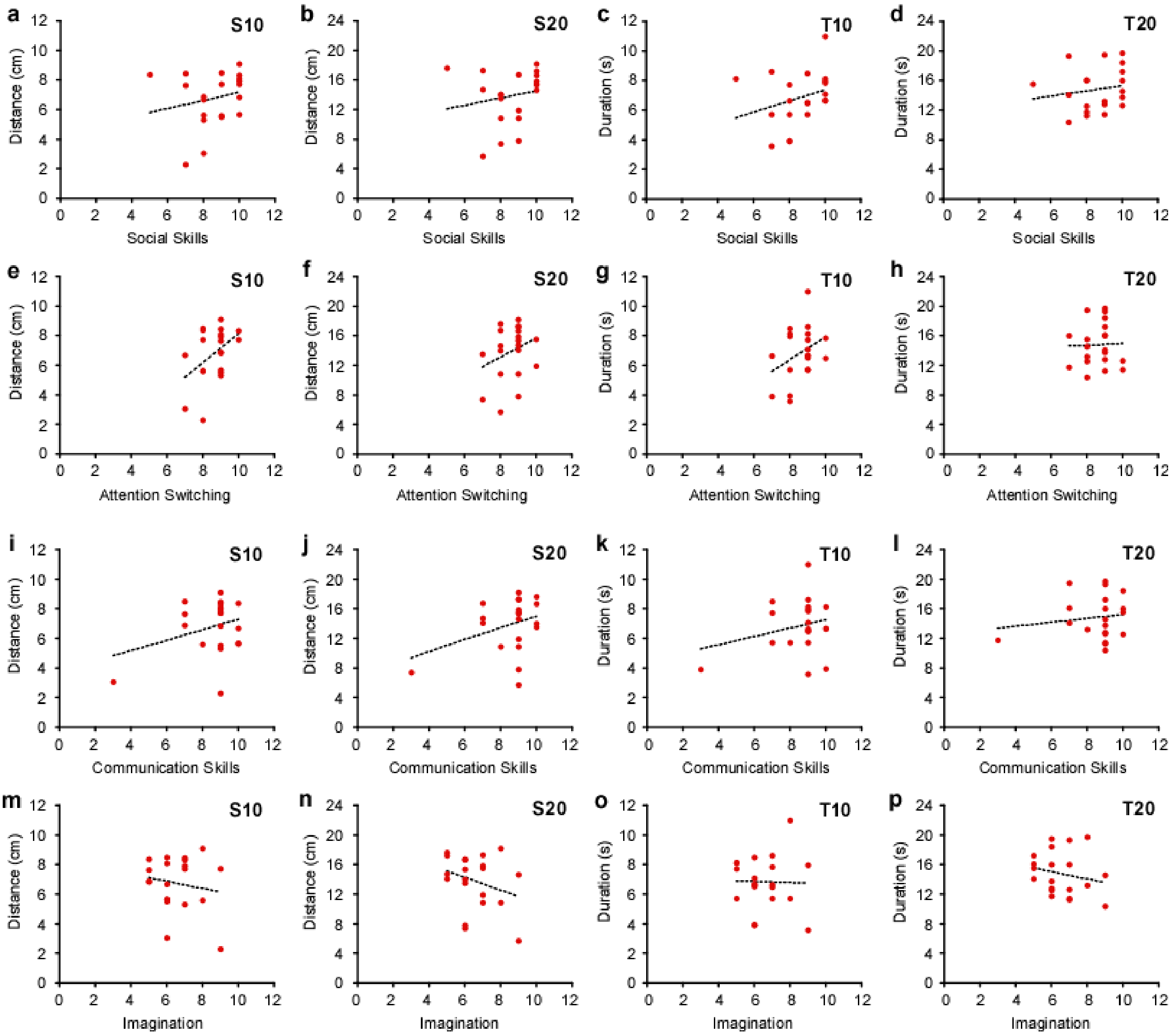

Supplementary Figure 3. Correlation between the AQ subscores and productions in the ASD group. Social skills with distance (**a, b**) and time duration (**c, d**). Attention switching with distance (**e, f**) and time duration (**g, h**). Communication skills with distance (**i, j**) and time duration (**k, l**). Imagination with distance (**m, n**) and time duration (**o, p**). All subscores showed no significant correlations for any of the conditions (see Supplementary Table 5).

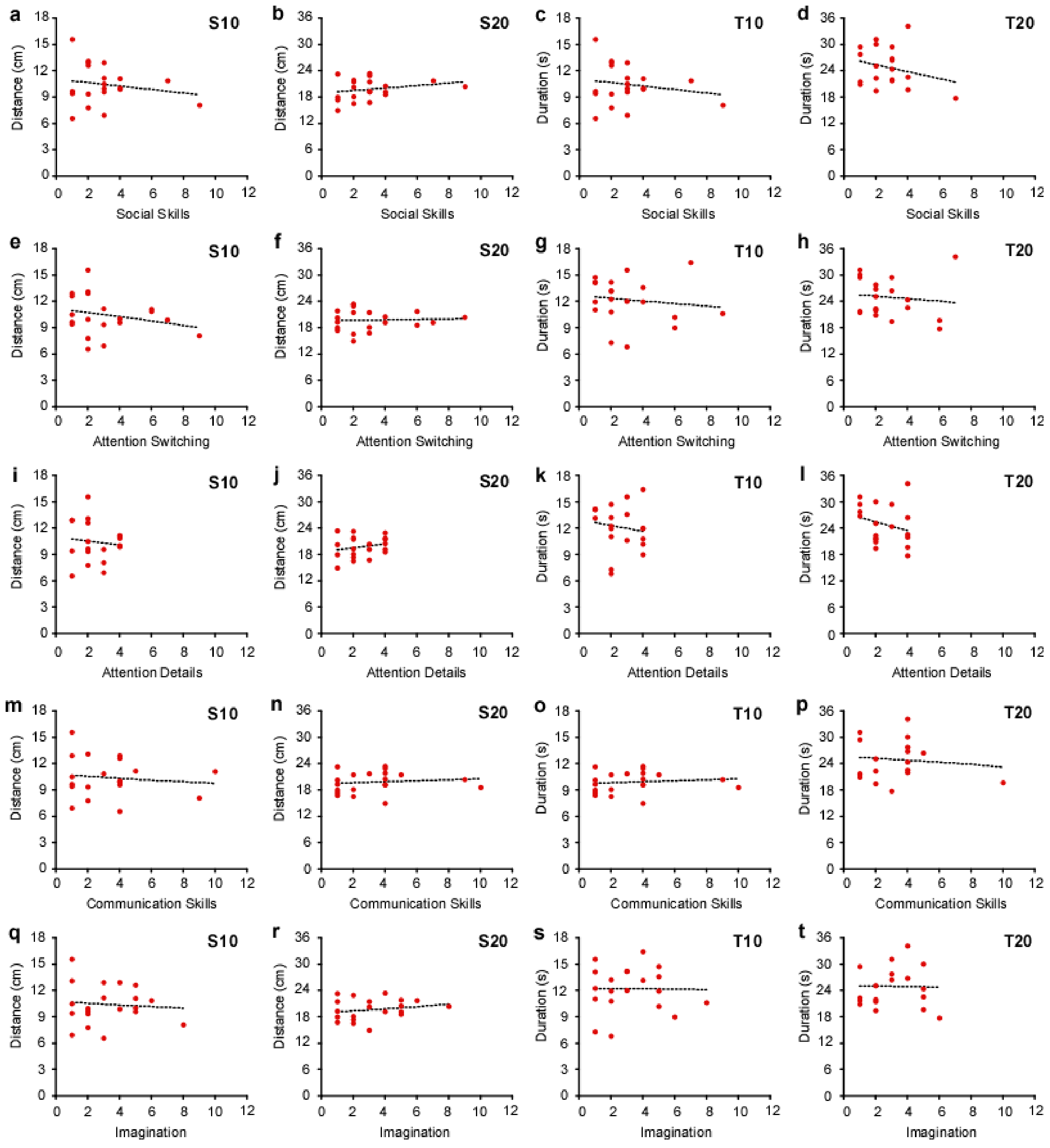

Supplementary Figure 4. Correlation between the AQ subscores and productions in the TD group. Social skills with distance (**a, b**) and time duration (**c, d**). Attention switching with distance (**e, f**) and time duration (**g, h**). Attention details with distance (**i, j**) and time duration (**k, l**). Communication skills with distance (**m, n**) and time duration (**o, p**). Imagination with distance (**q, r**) and time duration (**s, t**). All subscores showed no significant correlations for any of the conditions (see Supplementary Table 6).

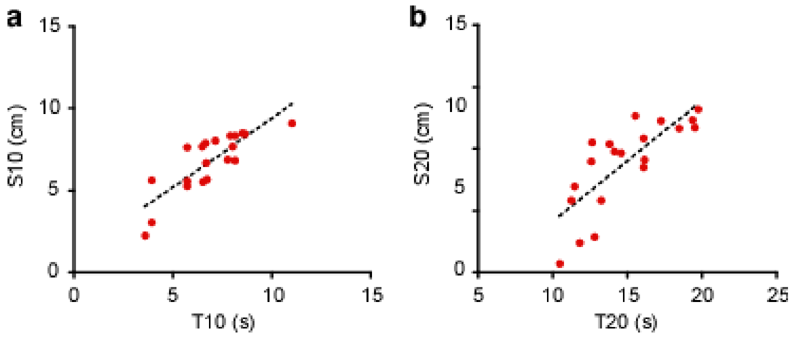

Supplementary Figure 5. The correlation between the distance and duration production. There were significant correlations between S10 and T10 (**a,** $r = 0.828$, $p < 0.0001$) and S20 and T20 (**b,** $r = 0.781$, $p < 0.0001$) in the ASD group (significance *p* value on FDR = 0.025).

Supplementary Video 1. Trial for the estimation of 10 cm (S10) without a cue (test session) in an individual with ASD. He estimated a shorter length in comparison to the predetermined length.

Supplementary Video 2. Trial for the estimation of 10 cm (S10) without a cue (test session) in a typical development (TD). He estimated a length close to the predetermined length without a cue.

Supplementary Video 3. Trial for the estimation of 10 cm (S10) with a ruler cue (feedback session) in an individual with ASD. He estimated a length close to the predetermined length along ruler.

Supplementary Video 4. Trial for the estimation of 10 cm (S10) with a ruler cue (feedback session) in a typical development (TD). He estimated a length close to the predetermined length along ruler.

Supplementary Video 5. Trial for the estimation of 20 cm (S20) without a cue (test session) in an individual with ASD. He estimated a short length in comparison to the predetermined length.

Supplementary Video 6. Trial for the estimation of 20 cm (S20) without a cue (test session) in a typical development (TD). He estimated a length close to the predetermined length without cue.

Supplementary Video 7. Trial for the estimation of 20 cm (S20) with a ruler cue (feedback session) in an individual with ASD. He estimated a length close to the predetermined length along ruler.

Supplementary Video 8. Trial for the estimation of 20 cm (S20) with a ruler cue (feedback session) in a typical development (TD). He estimated a length close to the predetermined length along ruler.

Supplementary Video 9. Trial for the estimation of10 s (T10) without a cue (test session) in an individual with ASD. He estimated a short duration in comparison to the predetermined duration.

Supplementary Video 10. Trial for the estimation of 10 s (T10) without a cue (test session) in a typical development (TD). He estimated a duration close to the predetermined duration without cue.

Supplementary Video 11. Trial for the estimation of 10 s (T10) with a clock cue (feedback session) in an individual with ASD. He estimated a duration close to the predetermined duration along clock.

Supplementary Video 12. Trial for the estimation of 10 s (T10) with a clock cue (feedback session) in a typical development (TD). He estimated duration close to the predetermined duration along clock.

Supplementary Video 13. Trial for the estimation of 20 s (T20) without a cue (test session) in an individual with ASD. He estimated short duration in comparison to the predetermined duration.

Supplementary Video 14. Trial for the estimation of 20 s (T20) without a cue (test session) in a typical development (TD). He estimated duration close to the predetermined duration.

Supplementary Video 15. Trial for the estimation of 20 s (T20) with a clock cue (feedback session) in an individual with ASD. He estimated duration close to the predetermined duration along clock.

Supplementary Video 16. Trial for the estimation of 20 s (T20) with a clock cue (feedback session) in a typical development (TD). He estimated duration close to the predetermined duration along clock.
